# Supplementary material for: The Artemiside-Artemisox-Artemisone-M1 Tetrad: Efficacies against Blood Stage P. falciparum Parasites, DMPK Properties, and the Case for Artemiside
Source: Pharmaceutics. 2021 Dec 3;13(12):2066. doi: 10.3390/pharmaceutics13122066 (PMC8704606; doi:10.3390/pharmaceutics13122066)
Supplement: Supplementary file 1 [file pharmaceutics-13-02066-s001.zip › pharmaceutics-1467552-Supplementary 1.pdf]

pharmaceutics

MDPI

Supplementary Materials: The artemiside-artemisox-artemisone-M1 tetrad: efficacies against blood stage P. falciparum parasites, DMPK properties, and the case for artemiside.

Liezl Gibhard, Dina Coertzen, Janette Reader, Mariëtte E. van der Watt, Lyn-Marie Birkholtz, Ho Ning Wong, Kevin T. Batty, Richard K. Haynes and Lubbe Wiesner

S1. Efficacy of artemisox: Dose-response curves of artemisox 6 against asexual blood stages and gametocytes of *Plasmodium falciparum* (*Pf*).

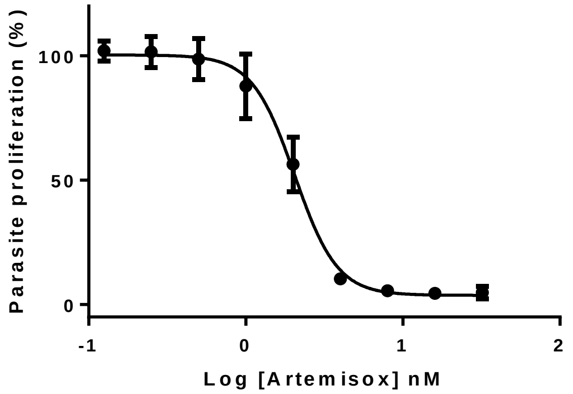

Figure S1a: Dose response curve of artemisox against the asexual blood stages of *Pf* NF54. The curve represents three independent biological repeats (n=3), ± SEM.

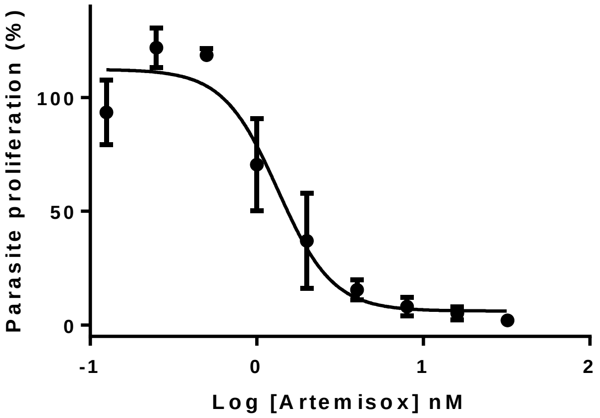

Figure S1b: Dose response curve of artemisox against the asexual blood stages of *Pf* K1. The curve represents three independent biological repeats (n=3), ± SEM.

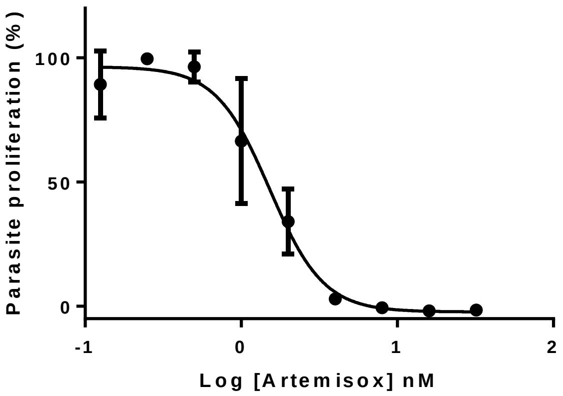

Figure S1c: Dose response curve of artemisox against the asexual blood stages of *Pf* W2. The curve represents three independent biological repeats (n=3), ± SEM.

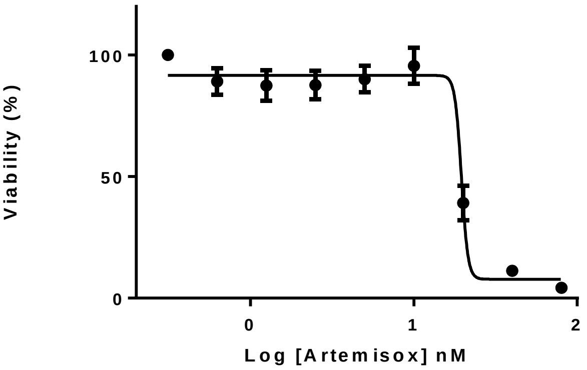

Figure S1d: Dose response curve of artemisox against early stage *Pf* NF54 gametocytes determined with the luciferase assay. Dose response was obtained at 48 h incubation. Each curve represents three independent biological repeats (n=3), ± SEM**.**

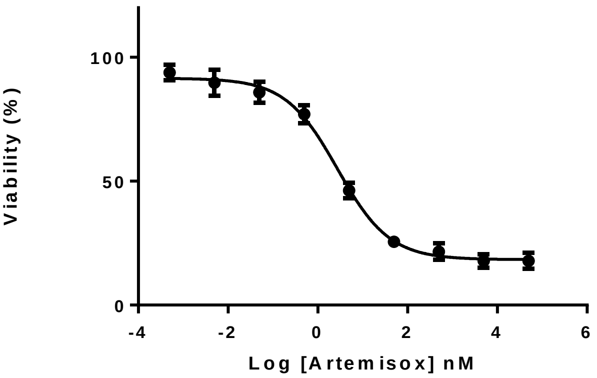

Figure S1e: Dose response curve of artemisox against late stage *Pf* NF54 gametocytes determined with the luciferase assay. Dose response was obtained at 72 h. Each curve represents three independent biological repeats (n=3), ± SEM.

S2. Efficacy of M1: Dose-response curves of M1 **8** against asexual blood stages and gametocytes of *Plasmodium falciparum*.

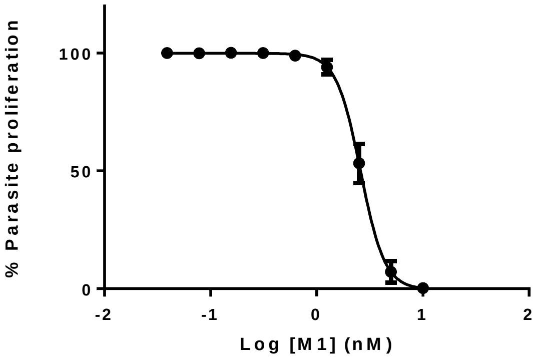

Figure S2a: Dose response curve of M1 against the asexual blood stages of *Pf* NF54. The curve represents three independent biological repeats (n=3), ± SEM.

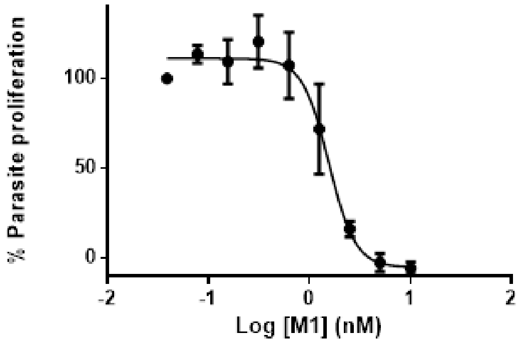

Figure S2b: Dose response curve of M1 against the asexual blood stages of *Pf* K1. The curve represents three independent biological repeats (n=3), ± SEM.

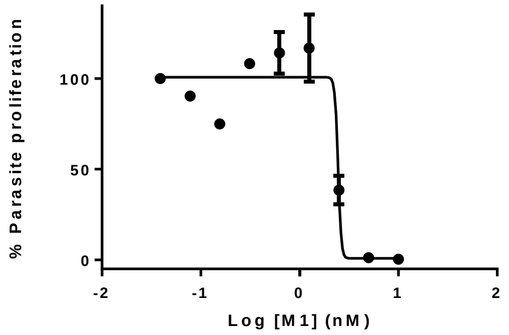

Figure S2c: Dose response curve of M1 against the asexual blood stages of *Pf* W2. The curve represents three independent biological repeats (n=3), ± SEM.

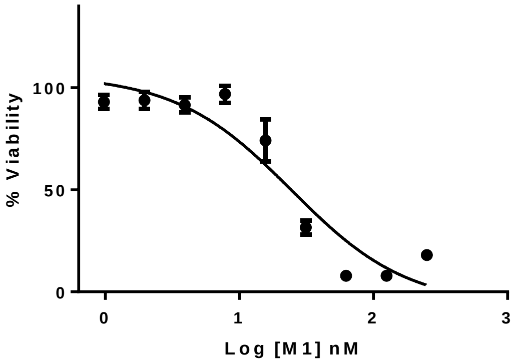

Figure S2d: Dose response curve of M1 against early stage *Pf* NF54 gametocytes determined with the luciferase assay. Dose response was obtained at 48 h incubation. Each curve represents three independent biological repeats (n=3), ± SEM**.**

S3. Pharmacokinetics and Metabolism: Circulating concentrations of each of artemiside **5**, artemisox **6** and artemisone **7** and respective metabolites (μM) after iv and po dosing of parent compounds in mice; iv = intravenous, po = oral; sd = standard deviation, blq = below limit of quantitation; n/a not available; n = no. of mice used.

Dosing of Artemiside 5

Table S3a: Concentrations of artemiside **5**

Time (h)	Mean iv (μM)	sd	n	Mean po (μM)	sd	n
0.08	6.241	0.914	3	0.085	0.027	3
0.25	3.555	1.020	3	0.109	0.004	3
0.5	1.711	0.348	3	0.135	0.014	3
1	0.930	0.281	3	0.119	0.008	3
3	0.180	0.088	3	0.058	0.023	3
5	0.086	0.036	3	0.012	0.002	3
7	0.037	0.014	3	0.006	0.001	2
24	0.008	0.003	3	blq	n/a	n/a

Table S3b: Concentrations of artemisox **6** after dosing of artemiside **5**

Time (h)	Mean iv (μM)	sd	n	Mean po (μM)	sd	n
0.08	1.926	0.400	3	1.864	0.260	3
0.25	1.817	0.400	3	2.150	0.382	3
0.5	1.376	0.339	3	2.510	0.282	3
1	0.674	0.388	3	2.026	0.183	3
3	0.066	0.017	3	0.286	0.228	3
5	0.020	0.001	3	0.007	0.001	3
7	0.011	0.004	3	0.005	0.000	1
24	0.005	0.000	2	blq	n/a	n/a

Table S3c: Concentrations of artemisone **7** after dosing of artemiside **5**

Time (h)	Mean iv (μM)	sd	n	Mean po (μM)	sd	n
0.08	0.117	0.015	3	1.003	0.051	3
0.25	0.247	0.068	3	1.387	0.177	3
0.5	0.270	0.093	3	1.786	0.135	3
1	0.160	0.102	3	1.671	0.104	3
3	0.012	0.007	3	0.179	0.137	3
5	0.005	0.000	1	blq	n/a	n/a
7	blq	n/a	n/a	blq	n/a	n/a
24	blq	n/a	n/a	blq	n/a	n/a

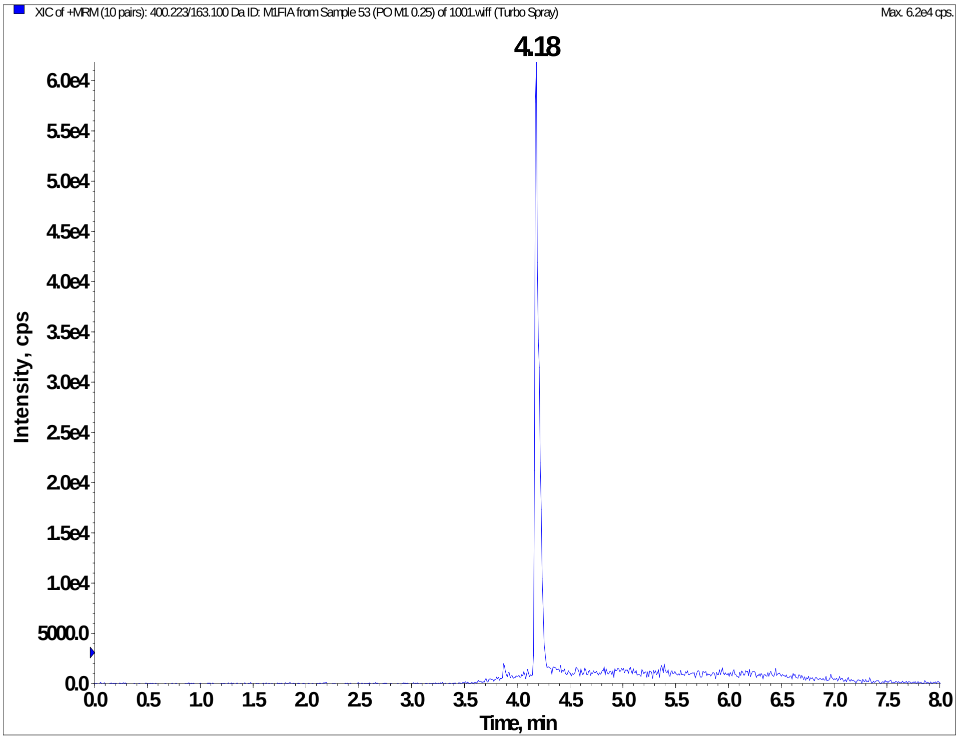

Figure S3a: LC-MS/MS chromatogram indicating presence of M1 following dosing of artemiside.[a]

[a]As determined by LC-MS/MS analysis with an AB SCIEX 5500 QTRAP instrument coupled with an Agilent 1260 HPLC detection system measured on mass of molecular ion $[M+H]^+$ (Transition 400.2/163.1) (ref. S1).

Dosing of Artemisox 6

Table S3d: Concentrations of artemisox **6**

Time (h)	Mean iv (μM)	sd	n	Mean po (μM)	sd	n
0.08	1.897	1.181	3	3.043	0.512	3
0.25	2.420	0.537	3	4.207	2.670	3
0.5	1.920	0.608	3	2.153	0.344	3
1	0.663	0.266	3	2.013	1.616	3
3	0.007	0.003	3	0.013	0.006	3
5	0.007	0.003	2	0.005	0.004	3
7	blq	n/a	n/a	0.004	0.001	2
24	blq	n/a	n/a	blq	n/a	n/a

Table S3e: Concentrations of artemisone **7** after dosing of artemisox **6**

Time (h)	Mean iv (μM)	sd	n	Mean po (μM)	sd	n
0.08	1.112	0.716	3	3.643	0.328	3
0.25	1.404	0.138	3	3.534	0.997	3
0.5	1.250	0.181	3	3.686	0.792	3
1	0.659	0.164	3	3.202	0.156	3
3	0.007	0.000	1	0.048	0.018	3
5	blq	n/a	n/a	0.007	0.000	1
7	blq	n/a	n/a	blq	n/a	n/a
24	blq	n/a	n/a	blq	n/a	n/a

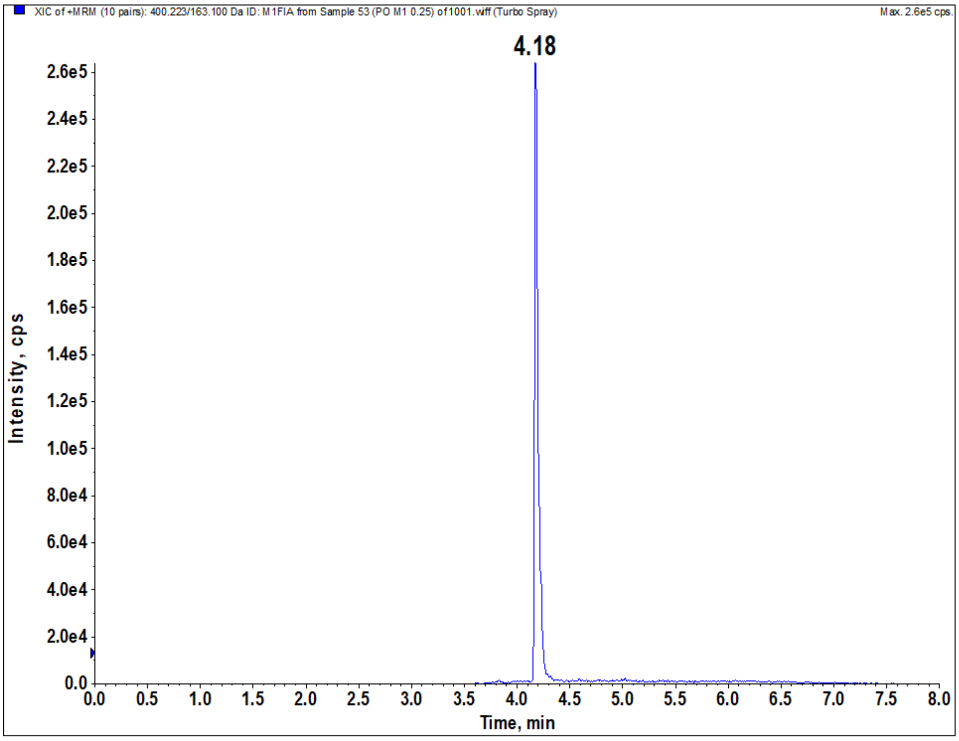

Figure S3b: LC-MS/MS chromatogram indicating presence of M1 following dosing of artemisox.[a]

[a] As determined by LC-MS/MS analysis with an AB SCIEX 5500 QTRAP instrument coupled with an Agilent 1260 HPLC detection system measured on mass of molecular ion $[M+H]^+$ (transition 400.2/163.1) (ref.S1).

Dosing of artemisone 7

Table S3f: Concentration of artemisone **7**

Time (h)	Mean iv (μM)	sd	n	Mean po (μM)	sd	n
0.08	3.864	0.854	3	1.626	0.466	3
0.25	2.506	0.691	3	1.523	0.163	3
0.5	1.715	0.389	3	1.135	0.249	3
1	0.831	0.374	3	0.357	0.096	3
3	0.028	0.021	3	0.012	0.010	3
5	blq	n/a	n/a	blq	n/a	n/a
7	blq	n/a	n/a	blq	n/a	n/a
24	blq	n/a	n/a	blq	n/a	n/a

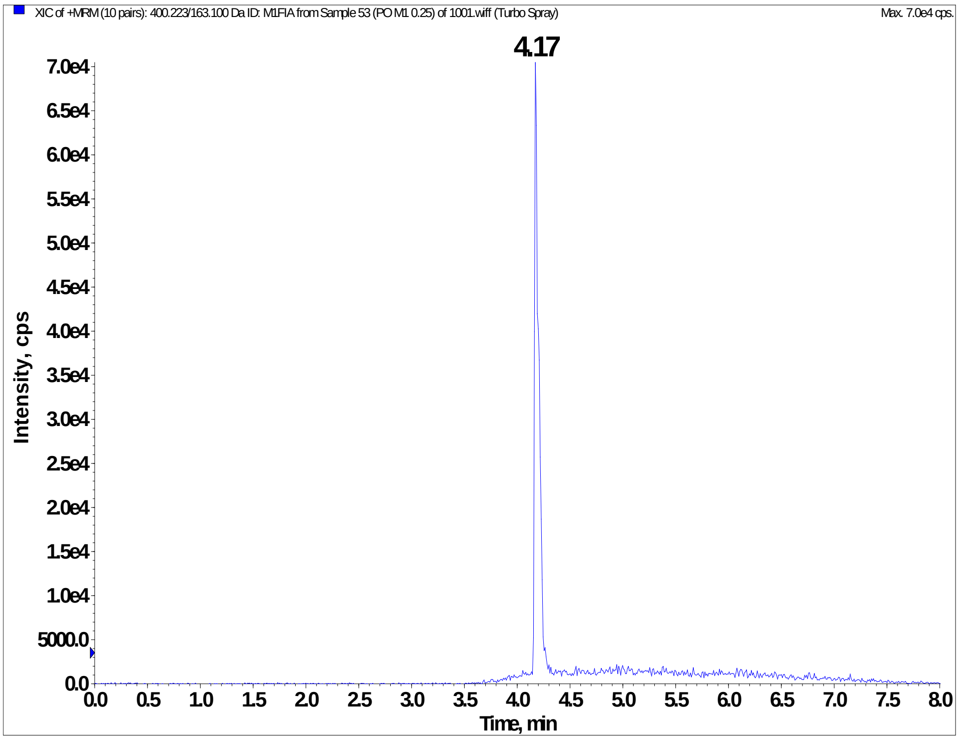

Figure S3c: LC-MS/MS chromatogram indicating presence of M1 following dosing of artemisone.[a]

[a] As determined by LC-MS/MS analysis with an AB SCIEX 5500 QTRAP instrument coupled with an Agilent 1260 HPLC detection system measured on mass of molecular ion $[M+H]^+$ (transition 400.2/163.1) (ref. S1).

S4. *In vitro* efficacy data (references S2, S3)

Table S4a: *In vitro* antimalarial activities for chloroquine, mefloquine, atovaquone, DHA **2**, artesunate **3**, artemisone **6**, metabolite M1 **12** determined with the [^{3}H]-hypoxanthine drug susceptibility assay (refs. S2).[a]

Compound	Antimalarial activities IC_{50} nM ± SEM					
	D6	W2	7G8	TM93-C1088	TM91-C235	TM-C2B
Chloroquine CQ	16 ± 2	195 ± 70	84 ± 18	360 ± 38	70 ± 12	95 ± 23
Mefloquine MFQ	ND	ND	5.4 ± 1.7	16 ± 5.0	107 ± 41	130 ± 51
Atovaquone	ND	ND	3.1±0.9	18,830 ± 5,102	2.2 ± 0.7	31,850 ± 6,833
DHA **2**	1.7 ± 0.4	2.2 ± 0.8	1.3 ± 0.2	1.4 ± 0.4	2.3 ± 0.7	2.0 ± 0.7
Artesunate **4**	ND	3.0 ± 1.6	1.5 ± 0.2	1.4 ± 0.1	2.9 ± 1.4	2.5 ± 0.5
Artemisone **7**	1.0 ± 0.4	1.3 ± 0.5	0.8 ± 0.1	0.7 ± 0.2	1.1 ± 0.5	1.1 ± 0.4
Metabolite M1 **8**	4.7 ± 0.2	6.6 ± 0.4	2.6 ± 0.8	2.5 ± 0.4	5.0 ± 0.6	8.6 ± 8.2

[a] D6 CQ sensitive; W2 and 7G8 CQ resistant; TM90-C2B and TM93-C1088 atovaquone and CQ resistant; TM91-C235 CQ and MFQ resistant; values represent the mean ± SD from three independent experiments carried out in triplicate

Table S4b: *In vitro* antimalarial activities of amino-artemisinins against *P. falciparum* asexual blood stage artemisinin-resistant clones carrying the *Pf*KI3 C580Y mutation as determined with the [^{3}H]-hypoxanthine drug susceptibility assay (ref. S3).

Compound [a]	W2		ARC08-22 (4G) (48 h lc)[b]			PL08-009 (5C) (36 h lc)[b]		
	IC_{50} nM	IC_{90} nM	IC_{50} nM	IC_{90} nM	RI[b]	IC_{50} nM	IC_{90} nM	RI[c]
DHA **2**	4.58±2.54	10.30±5.76	6.68±0.61	15.40±1.39	1.5	6.41±1.54	10.73±3.81	1.4
Artemiside **5**	2.21±0.42	4.28±0.25	2.43±0.13	4.85±0.09	1.1	0.29±0.03	0.43±0.03	0.1
Artemisone **7**	1.69±0.36	3.42±0.45	1.62±0.19	3.38±0.28	1.0	0.27±0.05	0.43±0.07	0.2

[a] Structures in Fig. 1; [b] lc = life cycle (h); [c] RI = IC_{50} for ARC08-22(4G)/IC_{50} for W2; [d] RI = IC_{50} for PL08-09 (5C)/IC_{50} for W2; Results are the mean of three independent biological replicates, performed as technical triplicates, ± SEM.

Table S4c: *In vitro* activities of selected amino-artemisinins against sporozoite liver stage *P. berghei* parasites and cytotoxicities (ref. S3)[a]

Compound	IC_{50} nM	Maximum inhibition % (Conc. μM)	Cytotoxicity EC_{50}[b]	
	P. berghei sporozoites		HepG2 μM	SI
Atovaquone	2.515±0.997	94.85±2.76 (0.5)	> 0.25	> 100
Puromycin	22.7±4.525	110±4.24 (5)	0.117	5.15
Artemether **3**	>10[4]	49.4 (10)	nd	nd
Artemiside **5**	81.3±9.616	99.05±1.34 (5)	> 25.0	> 308
Artemisone **7**	28.3±01.273	93.35±1.76 (10)	> 50.0	> 1767

[a] Structures in Fig. 1; luciferase-expressing *P. berghei* ANKA GFP-Luc-SM_{con} sporozoites were allowed to invade HepG2 cells and luciferase activity was measured after 48 h; data ± SD from biological duplicate and technical quadruplicate measurements. SI = EC_{50} HepG2/IC_{50} *P. berghei* sporozoites

S5. *In vivo* efficacy data from reference S4

Table S5: *In vivo* activities against CQ-sensitive *P. berghei* N strain and CQ-resistant *P. yoelii* NS strain (ref. S4).

Compound	*P. berghei* ED_{90} mg/kg		*P. berghei* artesunate index		*P. yoelii* ED_{90} mg/kg		*P. yoelii* artesunate index
	sc	*po*	*sc*	*po*	*sc*	*po*	*sc*
Artesunate **4**	7.2	7.1	1.0	1.0	22.0	-	1.0
Artemiside **5**	0.51	1.9	14.1	4.9	0.61	2.0	36.0
Artemisone **7**	1.5	3.1	3.2	2.3	3.9	5.0	5.6

[a]Peters four-day test with mice treated daily subcutaneously (sc) or orally (po) from the day of infection (day 0) through day 3; ED_{90} values are evaluated from parasite counts in peripheral blood on day 4; [b]ED_{90} artesunate/ ED_{90} compound.

S6. *In vitro* neurotoxicity data from reference S4 according to methods from refs. S5, S6

Table S6: Effects of the test compounds on viability and neurofilaments in rat primary neuronal brain stem cell cultures.[a]

Compound	Viability µg/mL[b]		ATP µg/mL[c]		Neurofilaments µg/mL[d]	
	NOEC[e]	IC_{50}	NOEC[e]	IC_{50}	NOEC[e]	IC_{50}
DHA **2**	0.1	5.0	0.01	0.08	<0.001	0.01
Artesunate **4**	0.1	>10	-	-	0.1	5.0
Artemiside **5**	1.0	>10	1.0	8.5	0.001	>10
Artemisone **7**	10	>10	>10	>10	>10	>10

[a]Determined on fetal rat brain stem cells (E18–E19) cultured to generate a permanent neuronal network during days 1–8; compounds in dimethyl sulfoxide were applied at 0.001, 0.01, 0.1, 1, and 10 mg/mL for 7 days starting on day 9 according to refs. S5,S6. [b]Cytotoxicity was measured by viability based on the activity of neuron-specific enolases. [c]ATP (adenosine triphosphate) levels measured at 1 day after initial administration (refs 5,6); [d]Neurotoxicity was assessed by the effect on the cytoskeleton on day 7; [e]No observable effect concentration.

References

S1 Watson, D.J., Laing, L., Gibhard, L., Wong, H.N., Haynes, R.K., Wiesner, L. Towards new transmission-blocking combination therapies - pharmacokinetics of 10-amino-artemisinins and 11-aza-artemisinin, and comparison with DHA and artemether. *Antimicrob. Agents Chemother,* **2021**, *65*, e00990-21, doi.10.1128/AAC.00990-21.

S2 Grobler, L., Chavchich, M., Haynes, R.K., Edstein, M.D., Grobler, A.F. Assessment of the induction of dormant ring stages in *Plasmodium falciparum* parasites by artemisone and artemisone entrapped in Pheroid vesicles *in vitro*. *Antimicrob. Agents Chemother.* **2014**, *58*, 7579-7582, doi: 10.1128/AAC.02707-14.

S3 Wong, H.N., Padín-Irizarry, V., van der Watt, M.E., Reader, J., Liebenberg, W., Wiesner, L. et al. Optimal 10-aminoartemisinins with potent transmission-blocking capabilities for new artemisinin combination therapies – activities against blood stage *P. falciparum* Including *Pf*KI3 C580Y mutants and liver stage *P. berghei* parasites. *Front. Chem.* **2020**, *7*, 901, doi: 10.3389/fchem.2019.00901.

S4 Haynes, R.K., Fugmann, B., Stetter, J., Rieckmann, K., Heilmann, H.- D., Chan, H.-W. et al. Artemisone - a highly active antimalarial drug of the artemisinin class. *Angew. Chem. Internat. Edit.* **2006**, *45*, 2082-2088, doi: 10.1002/anie.200503071.

S5 Schmuck, G., Haynes, R.K. Establishment of an in vitro screening model for neurodegeneration induced by antimalarial drugs of the artemisinin-type. *Neurotox. Res.* **2000**, *2*, 37-49. doi: 10.1007/BF03033326.

S6 Schmuck, G., Roehrdanz, E., Haynes, R.K., Kahl, R. Neurotoxic mode of action of artemisinin. *Antimicrob. Agents Chemother.* **2002,** *46*, 821 – 827, doi: 10.1128/aac.46.3.821-827.2002.
