# Supplementary material for: The Artemiside-Artemisox-Artemisone-M1 Tetrad: Efficacies against Blood Stage P. falciparum Parasites, DMPK Properties, and the Case for Artemiside
Source: Pharmaceutics. 2021 Dec 3;13(12):2066. doi: 10.3390/pharmaceutics13122066 (PMC8704606; doi:10.3390/pharmaceutics13122066)
Supplement: Supplementary file 1 [file pharmaceutics-13-02066-s001.zip › pharmaceutics-1467552-Supplementary 2.pdf]

The artemiside-artemisox-artemisone-M1 tetrad: efficacies against blood stage *P. falciparum* parasites, DMPK properties, and the case for artemiside.

Liezl Gibhard [1], Dina Coertzen [2], Janette Reader [2], Mariëtte E. van der Watt [2], Lyn-Marie Birkholtz [2], Ho Ning Wong [3], Kevin T. Batty [4], Richard K. Haynes [3*] and Lubbe Wiesner [5*]

1. H3D, Department of Chemistry, University of Cape Town, Observatory, 7925, Cape Town, South Africa.
2. Malaria Parasite Molecular Laboratory, Department of Biochemistry, Genetics and Microbiology, Institute for Sustainable Malaria Control, University of Pretoria, Pretoria, South Africa.
3. Centre of Excellence for Pharmaceutical Sciences, Faculty of Health Sciences, North-West University, Potchefstroom, 2520, South Africa
4. Curtin Medical School, Curtin University, Bentley, Western Australia, 6102.
5. Division of Clinical Pharmacology, Department of Medicine, University of Cape Town, Observatory, 7925, Cape Town, South Africa

Supplementary Material 2

Copy of reference 37 main text: Schmuck, G.; Temerowski, M.; Haynes, R.K.; Fugmann, B. Identification of non-neurotoxic artemisinin derivatives in vivo and in vitro. *Res. Adv. in Antimicrob. Agents and Chemother.* **2003**, *3*, 35-47.

Copy of reference 37 main text: Schmuck, G.; Temerowski, M.; Haynes, R.K.; Fugmann, B. Identification of non-neurotoxic artemisinin derivatives in vivo and in vitro. *Res. Adv. in Antimicrob. Agents and Chemother.* **2003**, *3*, 35-47.

Res. Adv. in Antimicrob. Agents & Chemother. 3, 2003

Identification of non-neurotoxic Artemisinin-derivatives *in vivo* and *in vitro*

Schmuck Gabriele*, Michael Temerowski, Richard Haynes*** and Burkhard Fugmann******

*BAYER AG, Pharma Research Centre, Aprather Weg, D-42096, Wuppertal, Germany; E-mail: GABRIELE.SCHMUCK.GS@bayer-ag.de; **Bayer AG, Crop Science, Aprather Weg, Wuppertal, Germany; E-mail: michael.temerowski.mt@bayer-ag.de; ***Department of Chemistry, The Hong Kong University of Science and Technology, Clear Water Bay, Kowloon, Hong Kong; E-mail: haynes@ust.hk; ****Bayer AG, BBS-IM-RDS, Building Q 18, Leverkusen, Germany;
E-mail: burkhard.fugmann.bf@bayer-ag.de

ABSTRACT

The establishment of an *in vitro* screening model for neurodegeneration inducing antimalarial drugs was conducted in stepwise fashion. Firstly, investigations in human and rodent differentiated neuronal cell cultures (Kelly, N-18) showed that the sensitivity was not sufficient for a screening system. Secondly, a comparison between different parts of the brain (cortex vs. brain stem) identified the brain stem to be more sensitive than the cortex. The neurotoxic potential was determined by cytoskeleton elements (neurofilaments), which were degradated *in vitro* by diverse neurodegenerative compounds. In comparison of dog and rat primary brain stem cultures, the dog cells were found to be more sensitive to artemisinin than the rat cells. However, from an ethical point of view, dog primary cell cultures were not applicable.

A battery of new drug candidates out of an antimalarial cooperation between Bayer AG and Hongkong University of Science and Technology were tested in the rat brain stem assay. For evaluation of in vivo relevance, a drug candidate named artemisone,-identified to be non-

*Author to whom correspondence should be addressed.

neurotoxic *in vitro*, was compared to another, but neurotoxic drug candidate *in vivo* and to artesunate, the standard drug on the market. Rats were treated with these compounds in parallel by gavage with the dosages 10 and 50 mg/kg bodyweight over 14 days. During this time course a daily functional observation battery (FOB) was performed to identify neurotoxic symptoms. It could be demonstrated, that artemisone was non neurotoxic at a dosage of 50 mg/kg bodyweight *in vivo* in contrast to artesunate and candidate, which was highly neurotoxic. In conclusion, the establishment of a sensitive screening system *in vitro* using rat brain stem neuronal cell cultures and *in vivo* allowed the selection of potential antimalarial drug candidates of the artemisinin type with no neurotoxic potential *in vitro* and *in vivo*

INTRODUCTION

The search for new antimalarial drugs has been handsomely rewarded in recent times with the isolation from the traditional Chinese herb *Artemisia annua L.*, and characterization of the sesquiterpene qinghaosu (artemisinin) in China. Artemisinin is remarkable for being the first representative of a naturally occurring compound class containing the 1,2,4-trioxane structural unit [1,2,3]. Artemisinin, and its derivatives dihydroartemisinin, artemether and artesunate (Fig. 1), are effective blood schizonticidal agents against *Plasmodium falciparum* and other *Plasmodium* species, with activity of the drugs being conferred by the embedded 1,2,4-trioxane. As reflected in the fact that over two million patients have been treated acceptance of the drugs by malaria clinicians is now widespread. [4,5,6,7.8,9]. The drugs are the most rapidly acting of any antimalarial drug in inducing clearance of parasitemia, and are the most effective in reducing the time to awakening of patients in malaria coma [9,10.

Animal studies in dogs and rats indicated, that these compounds may have a considerable neurotoxic potency. Multiple dose studies with arteether, which is degraded to the active metabolite dihydroartemisinin, in beagle dogs showed a clear dose dependent neurotoxicity of the compound (5-20 mg/kg/day). All high dose animals displayed a progressive syndrome of clinical neurologic defects with progressive cardiorespiratory collapse and death in five of six animals [11]. Neurologic findings included gait disturbance, loss of spinal and pain response reflexes, a prominent loss of brain stem and eye reflexes. Prominent neuropathic lesions were noted to be restricted primarily to the pons and

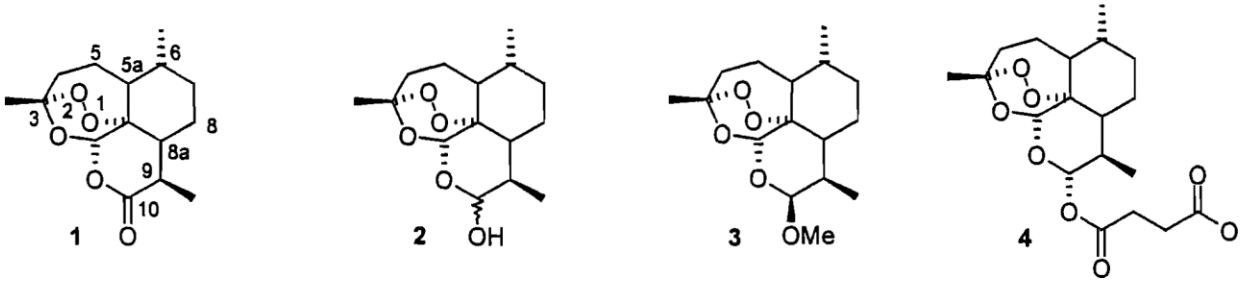

Fig. 1: Artemisinin and its derivatives: **1** = artemisinin (qinghaosu); **2** = dihydroartemisinin; **3** = artemether; **4** = artesunate

medulla. The same observations could be made in a 28 day rat study at doses of 12.5-50 mg/kg/day [12]. *In vivo* toxicity studies in the rat and monkey also reveal neuropathies in the caudal brain stem [13,14,15,16]. The histological changes have been found to occur in the absence of neurological signs or behavioural performance deficits.

In humans treated with the artemisinin drugs, adverse affects directly ascribed to neurotoxicity have yet to be unambiguously demonstrated, but may possibly be due to neurodegeneration (3.3% of the cases) [2,5,8,17]

To summarize, whilst positive neurological effects have yet to be observed in patients treated with artemisinin drugs, the results of the *in vitro* and animal *in vivo* assays for neurotoxicity represent a severe bar to registration of artemisinin derivatives by Western drug regulatory authorities.

Recently, we published a new *in vitro* screening method using primary brain stem cell cultures from the rat [18]. We could demonstrate that the brain stem cells were selectively sensitive against artemisinin in contrast to another brain region the cortex. Endpoints in this *in vitro* assay were cytotoxicity measured by the viability assay *(live/dead assay)* and a neuronal cytoskeleton marker, the 200 kDa neurofilaments. Artemisinin diminished the amount of non-phosphorylated neurofilaments specifically below the cytotoxic range and it was postulated that the cytoskeleton may be a sensitive target for this specific neurodegeneration in the brain stem. Comparable effects were shown with 3 other derivatives dihydroartemisinin, artemether and artesunate. The ranking between these 4 compounds *in vitro* was comparable to the *in vivo* situation [12].

Mechanistic investigations using cortical and brain stem neuronal cell cultures demonstrated the selective toxicity of artemisinin on brain stem cell cultures. It was shown with biochemical and molecular biological methods that oxidative stress by artemisinin is responsible for the neurodegenerations induced in animals [19].

A testing battery of many different artemisin derivatives, was necessary to identify one compound, which showed no neurotoxic potential *in vitro*. However, these findings must be verified by a repeated dose experiments in rats using the standard compound on the market artesunate, a neurotoxic candidate *in vitro* and artemisone, which was shown to be not neurotoxic *in vitro*. The screening system in rats included beside the observations of clinical signs a behavioral test battery with the few on neurotoxicity.

MATERIAL AND METHODS

Cell Culture of Primary Cells

The brain stems were dissected from the whole brain tissue of fetal rats in the developmental stage E 18 -E 19 and subsequently ensheated from the cerebral membrane. The tissues were pooled in sterile cultivation medium (Neurobasal medium, Invitrogen, Heidelberg, Germany) containing 10 mL B27 (Invitrogen, Heidelberg, Germany). The brain stem was digested with Collagenase A (Boehringer, Ingelheim, Germany) (150 mg/50 ml medium) for 30 min. The isolation of individual cells from brain stem tissues was performed by filtration of the neuronal cells through two Nylon meshes with different pore diameters (135 and 22 µm). The single cell suspension was centrifuged (500-700 g) and washed twice with culture medium. The cell pellet was then suspended in 10 ml of the culture medium and the cell number counted by a cell analyzer system (Schärfe System, Reutlingen, Germany). The cells were transferred in a cell concentration of 5 x 10^5 to 1 x 10^6 cells per well in 24 well poly-d-lysine coated cell culture plates (Biocoat Becton and Dickinson, Heidelberg, Germany).

As neuronal cell cultures generate a permanent neuronal network within 8 days, the test procedure commenced at day 9 and was completed at day 16. During this time, the cell composition did not change because the exclusion of the serum inhibited glial growth [18,19These cultures are highly reproducable using the above described, serum-free conditions. This was also documented by statistical analysis of the results.

The cell cultures were treated in

concentrations between 0.001 – 10 µg/ml dihydroartemisinin, artesunate, artemisone and candidate solved in DMSO (Stock solution 10 mg/ml). DMSO controls were run in parallel to a non-treated control. 0.1 % DMSO in maximum had no effect on the cell cultures (data not shown). Each experiment was repeated three times with three different cell preparations.

Viability assay (live/dead assay)

The viability assay was used in the sprouting assay and in primary cell cultures. Cells were eluted twice with PBS and subsequently incubated in a Calcein-AM/PBS solution (1:2) (Molecular Probes, Eugene U.S.A.) for 30 minutes in a cell incubator. Fluorescence was determined with a Fluostar spectrophotometer (SLT, Crailsheim,Germany) at 485/530 nm.

ELISA

The cell culture plates were fixed in cold methanol (4 °C) for 10 minutes and subsequently incubated for one hour in a 0.1% human albumin/PBS solution (Sigma, Deisenhofen, Germany). The cells were then treated with detergent (0.3% Triton X 100 in PBS; Sigma., Deisenhofen, Germany) for 10 minutes and then eluted two times with PBS (+ 0.3% gelatin; Sigma, Deisenhofen, Germany). The first antibody (neurofilament (non-phosphorylated; 200 kDa; (1:100); mouse; Roche, Ingelheim, Germany) was added for 2 hours, the second antibody (anti- mouse (1:500), Roche, Ingelheim, Germany) for 1 hour at 4 °C. After removal of antibody 1 and 2, the plates were washed three times with PBS (+ 0.3 % gelatin). The attached 2. antibodies were coupled to horse radish peroxidase and were quantified by a peroxidase substrate, the ABTS-solution (Roche, Ingelheim, Germany) for 30 minutes. The quantification of the attached antibodies was conducted at 405 nm in an ELISA reader.

Animal studies

Various derivatives were administered by gavage to 2 male Wistar rats per group, aged approximately 8 weeks using doses of 10 and 50 mg/kg body weight for 14 days.

Analysis of test compound in the administration formulation was not performed. The test compound required for every week was solubilized in sesame oil at the start of the study and after one week of treatment. Prior to application the formulations were well mixed by shaking the glass tubes and by pumping the syringe several times.

Animals were weighed daily and were observed several times per day for clinical signs. In addition, animals were placed into an open field two times per day in order to observe the animals for piloerection, respiratory abnormalities,

posture, involuntary motor movements, stereotypy, bizarre behavior, gait abnormalities, vocalizations, arousal, rearing, defecation and urination. Manipulative testing such as reaction and reflex testing was also planned in the original protocol but it was dropped prematurely because it turned out, that „gait abnormalities“ and „body weight development“ were already very sensitive measures for the identification of major endpoints of concern. Animals were necropsied after exsanguination under deep ether anesthesia.

Statistical analysis

The statistics for the cell culture assays was performed in a two step procedure: a) ANOVA; b) t-test (Student-Newman-Keuls methods) (Sigma Stat; Scientific, Erkrath, Germany). The primary cell cultures from the rat came from 3 separate preparations with 4 replicates in each experiment.

RESULTS

Cytotoxicity in primary neuronal brain stem cells

The cytotoxicity was measured by a viability assay which determines unspecific esterases. Artesunate and candidate were highly cytotoxic to brain stem cell cultures with a NOEC of 0.01 µg/mL and EC_{50} values of 5 and 4.8 µg/mL, respectively. Dihydroartemisinin was less cytotoxic with a NOEC of 0.1 and an EC_{50} value of > 10 µg/mL. However, artemisone showed no cytotoxicity at all up to 10 µg/mL (fig. 2).

Neurotoxicity in primary neuronal brain stem

As a more specific endpoint, the cytoskeleton was determined because artemisinin and its derivatives induce neurodegenerations in the brain stem for which the cytoskeleton elements is a surrogate.

For the endpoint neurofilaments, no NOEC levels in rat brain stemm cells could be determined at the lowest concentration used for dihydroartemisinin and candidate of 0.001 µg/mL. The EC_{50} values were 0.04 µg/mL for dihydroartemisinin and 2.2 µg/mL for candidate. Here artesunate has weakest effects with a NOEC level of 0.1 µg/mL and an EC_{50} value of 5 µg/mL. However, artemisinone showed no effects on neurofilaments up to a concentration of 10 µg/mL (fig. 3).

Neutotoxicity screening battery in vivo

Objective of the study was, to determine the systemic tolerance of male rats to

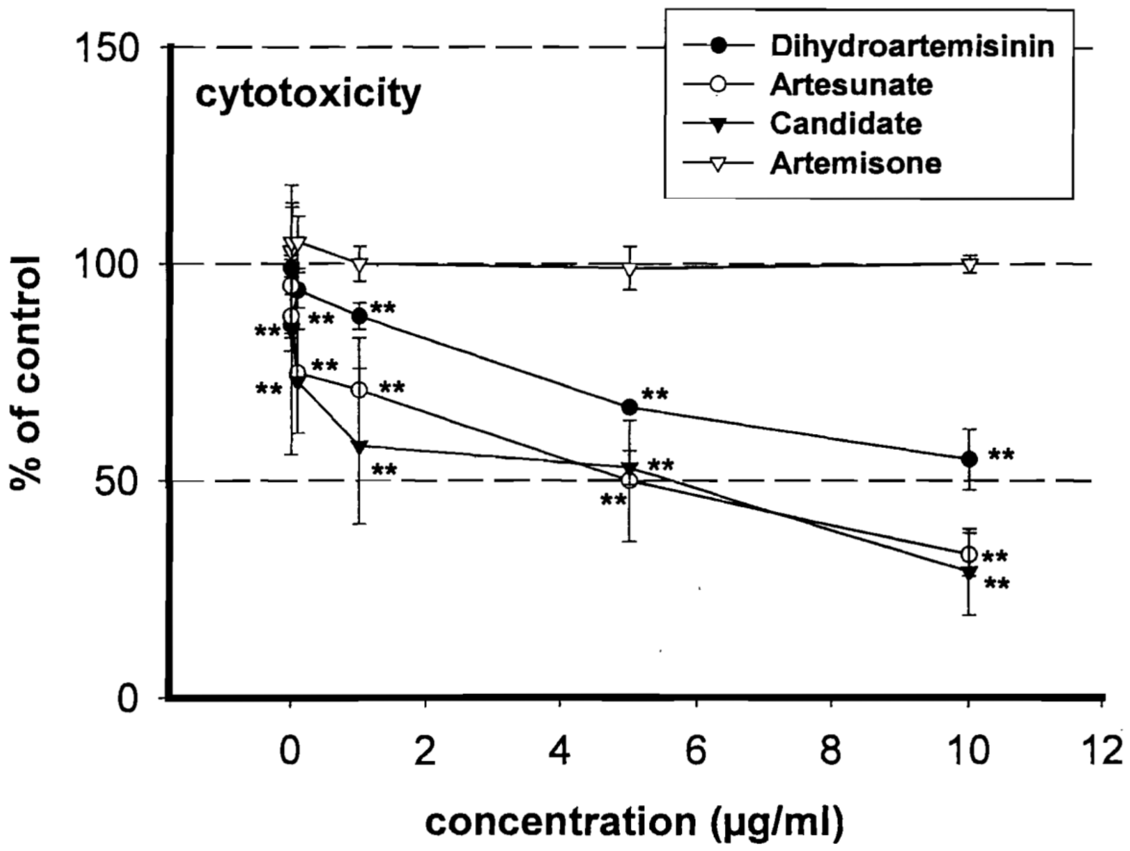

Fig. 2: Determination of the cytotoxicity of dihydroartemisinin, artesunate, artemisone and candidate on primary brain stem cell cultures after treatment over a period of 7 days: (** $p \leq 0.01$).

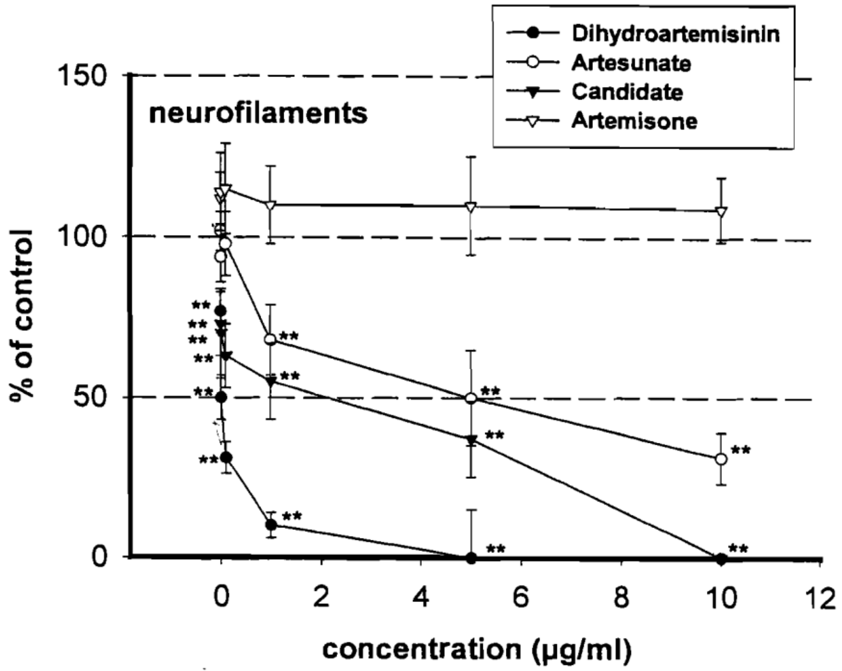

Fig.3: A comparison was made with 4 compounds: dihydroartemisinin, artesunate, artemisone and candidate. Brain stem cell cultures of the rat were used and neurofilaments were detected as a specific endpoint for neurodegenerations. They were treated with the compounds of 7 days. (** $p \leq 0.01$).

Table 1: Behavioral symptoms in the open field

(- = no; + = slight; ++ = moderate; +++ = severe)

**Compound**	**Symptoms**
Artesunate	+
Artemisone	-
Candidate	+++

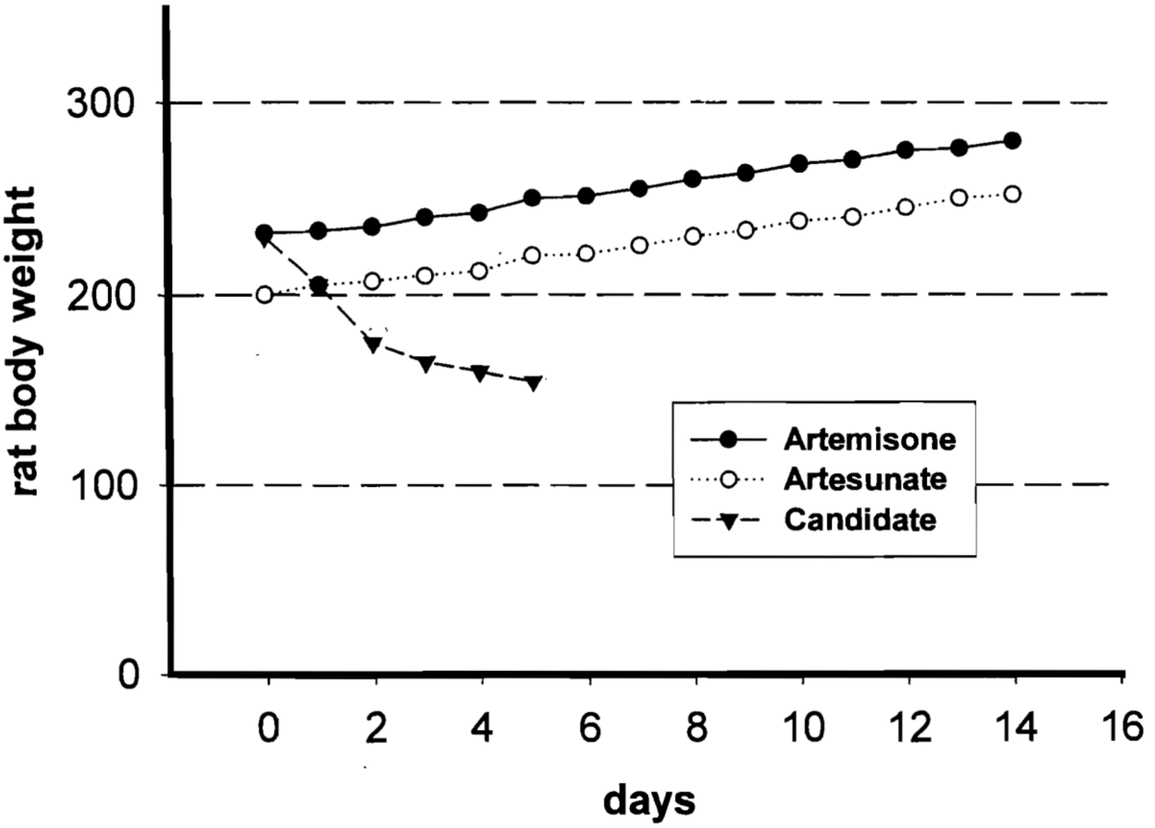

Fig. 4: Body weight development of male rats treated with 50 mg/kg bw. artesunate, artemisone or candidate over 14 days.

exposure of various artemisinin-derivatives and to identify possible neurologic findings associated with the exposure.
The rats were treated orally with 10 or 50 mg/kg bw. artesunate, artemisone and candidate over 14 days. The animals were weight daily and were observed for clinical signs several times per day in the home cage and in the open field. The body weight development was shown in fig. 4, the behavior was summarized in table 1.
Animals treated with artesunate, receiving 50 mg/kg bw. revealed an uncoordinated gait from day 7 to day 11. Furthermore, vocalizations were noticed from day 7 to day 10. The

average body weight gain within the 14 days of treatment was 63 g for the low dose group and 61 g for the high dose group, which is slightly lower when compared with historical controls (80 g).

Animals treated with candidate (50 mg/kg bw.) showed severe body weight loss, reduced motility, uncoordinate gait, diarrhoea and piloerection. Both animals died after 5 or 6 days of treatment.

No effects on body weight, clinical signs and neurobehavior were observed with artemisone. The compound was well tolerated during the 14 days treatment period.

In summary, body weight loss, uncoordinated gait and reduced motility were the most prominent clinical signs found with various artemisinin derivatives (data not shown).

DISCUSSION

The use of neuronal cells from the brain stem clearly demonstrated that artemisinin and derivatives act selectively on differentiated neuronal cell cultures with well developed sprouts quantified by neurofilaments (200 kDa) [18]. Differentiated cell cultures built up a complicated network of neurites, which made them more sensitive to neurodegenerative effects. This has been shown for organophosphates [20] as well as for other neurotoxins like acrylamide and n-hexane [21,22]. However, it could be demonstrated by Fishwick et al. [23] that dihydroartemisinin did not affect the neurofilaments directly. A reduction of the neurits and in parallel a degradation of the neurofilaments occured by other mechanisms like an inhibition of respiratory functions or oxidative stress. It is known from inhibitors of the respiratory chain like KCN that they induce delayed Parkinsonism and a basal ganglia disease which is associated with lokomotor impairment [24,25,26]. However, it was shown in the same test system using cortical neurons that KCN induced no direct disturbance of the neurofilaments in contrast to paraquat a model compound for oxidative stress [22,27]. Therefore, investigations were done with artemisinin in cortical and brain stem cell cultures to clarify the underlying mechanism for ist neurodegenergations. Consideration of all available data together makes it seem likely that the neurotoxic mechanism of artemisinin is related to the possibility to produce radicals and, in parallel, to inhibit the respiratory chain. Neuronal cells seemed to be generally more sensitive than non- neuronal cells because their antioxidative defense system is less active [27]. An interference with the respiratory chain induce irreversible degenerations as

shown with other neurotoxins. The differences between the brain areas may be related to kinetic variations because the cortical cells showed clear neurotoxic effects after a longer time period [19].

In patients, only few adverse effects were reported (3.3% of the cases) [2,5,8,17]. Also dihydroartemisinin and artemether were considered as well tolerated and as a possible alternative to quinine. The species selectivity could not be based on bioviability of the drug. Dihydroartemisinin reached the highest plasma levels in humans [28] followed by the rat and the lowest levels were reached in dogs and rabbits [29]. Half-lives were 2 h in humans, 1-2 h in dogs and 30 min. in rats [29,30,31]. One plausible explanation for the species sensitivity were given by Ashton et al., [30] which described a marked sex differences in rats based on a different liver P 450 enzyme pattern in male and in female rats. These variations very also obvious between species.

In summary, the neurotoxic potential of artemisinin and its derivatives can be demonstrated in a specific *in vitro* model like the rat brain stem cell culture. In parallel, efficacy testing in infectious animal models and a verification of the toxicity in a specific neurotoxicity test battery in rats is necessary to identify an optimal candidate for human use

ACKNOWLEGMENT

We than the Medicine for Malaria Venture (MMV), Geneva, Switzerland, for financial support.

REFERENCES

1. Klayman, D. L. (1985) Science 228, 1049-1055

2. Looareesuwan, S.; Wilairatana, P.; Vanijanonta, S.; Pitisuttithum, P.; Viravan, C. (1996) Annals of Tropical Medicine and Parasitology 90, 21-28

3. Woerdenbag, H. J.; Lugt, C. B.; Pras, N. (1990) Pharm. Weekbl. [Sci.] 12, 169 - 181.

4. Cao, X. T.; Bethell, D. B.; Pham, T. P.; Ta, T. T.; Tran, T. N.; Nguyen, T. T.; Pham, T. T.; Nguyen, T. T.; Day, N. P., White, N. J. (1997) Transactions of the Royal Society of Tropical Medicine & Hygiene. 91(3), 335-342

5. Hien, T. T.; Day, P. J. N; Phu, N. H.; Hoang, N. T.; Chau, T. T. H.;

Loc, P. P.; Sinh, D. X.; Cuong, L. V.; Vinh, H.; Waller, D.; Peto, T. E. A.; White, N. J. (1996) *N.* Engl. J. Med. 335, 76-83.

6. Hien, T. T.; White, N. J. (1993) Lancet 341, 603.

7. Price, R. N.; Nosten. F.; Luxemburger, C.; van Vugt, M.; Phaipun, L.; Chongsuphajaisiddhi T.; White, N. J. (1997). Transactions of the Royal Society of Tropical Medicine & Hygiene. 91(5), 574-577.

8. van Hensbroek, M. B.; Onyiorah, E.; Jaffar, S.; Schneider, G.; Palmer, A.; Frenkel, J.; Enwere, G.; Forck, S.; Nusmeijer, A.; Bennet, S.; Greenwood, B.; Kwiatowski, D. (1996) N. Engl. J. Med. 335, 69-75.

9. White, N. J. (1994) Trans. Roy. Soc. Trop. Med. Hyg. 88,Supplement 1 S3-S4

10. Arnold, K. (1993) J. Hong Kong. Med. Assoc. 45, 189-196.

11. Phillips-Howard, P.; ter Kuile, F. O. (1995) Drug safety 12, 370-383

12. Brewer. T.G.; Genovese,R.F.; Newman, D.B.; Li, Q. (1998). Medicine Tropicale 58 Suppl. 3, 22S-27S

13. Genovese, R. F.; Newman, D. B.; Li, Q.; Peggins, J. O.; Brewer, T. G. (1998) Brain Research Bulletin 45, 199-205

14. Genovese, R. F.; Petras, J. M.; Brewer, T. G. (1995) Annals of Tropical Medicine & Parasitology 89(4), 447-449.

15. Petras, J. M., D. E. Kyle, M. Gettayacamin, G. D. Young, R. A. Bauman, H. K. Webster, K. D. Corcoran, J. O. Peggins, M. A. Vane, T. G. Brewer. (1997). American Journal of Tropical Medicine & Hygiene **56:** 390-396.

16. Petras, J. M., G. D. Young, R. A. Bauman, D. E. Kyle, M. Gettayacamin, H. K. Webster, K. D. Corcoran, J. O. Peggins, M. A. Vane, T. G. Brewer. (2000). Anat. Embryol 201: 383-397

17. Price.R.; Van Vugt,M.; Phaipun, L.; Luxenburger, C.; Simpson, J., McGready, R.; Ter Kuile, F.; Kham, A.; Chongsuphajaisiddhi, T.; White, N.T.; Nosten,F. (1999). Am. J. Trop.Med. Hyg. 60: 547-555

18. Schmuck G. & R. Haynes. (2000) Neurotoxicity Research 2: 37-49.

19. Schmuck G, E. Röhrdanz, R.K. Haynes & R. Kahl. (2001). Antimicrobial. Antimicrobial Agents Chemother 46:821-827

20. Schmuck, G.; Ahr, H.-J. (1997) Toxicology in vitro 11, 263-270

21. Schmuck, G.; Schlüter, G. (1996) Toxicology and Industrial Health 12, 683-696

22. Schmuck,G.; Ahr,H.J.; Schlüter,G. (2000) In vitro and molecular Toxicology 13: 37-50

23. Fishwick,J.; Edwards,G.; Ward,S.A. McLean, W.G. (1998b) NeuroToxicology 19, 393-404

24. Dawson,R.; Beal,M.F., Bondy,S.C., Di Monte, D.A., Isom, G.E. (1995) Toxicol. Appl. Pharmacol. 134, 1-17

25. Kanthasamy,A.G.; Rathinavelu,A.; Borowitz,J.L.; Isom,G.E. (1994) NeuroToxicology 15, 887-896

26. Krieglstein, J.; Nuglisch,J. (1992) In: Herken,H.; Hucho,F. (eds.), *Selective Neurotoxicity* (Springer Verlag) pp.111-140

27. Schmuck G, E. Röhrdanz, R.K. Haynes & R. Kahl. (2001). Antimicrobial. Antimicrobial Agents Chemother 46:821-827

28. Zhao K.,C.; Song Z.,Y. (1993). Yao Xue Xue Bao;28(5):342-6

29. Li,Q.; Peggins,J.O.; Fleckenstein,L.L., Masonic,K.; Heifer,M.H.; Brewer,T.G. (1998) J. Pharm. Pharmacol. 50, 173-182

30. Ashton,M.; Johansson,L.; Thornqvist,A.-S.; Svensson,U.S.H. (1999) Xenobiotica 29, 195-204

31. Dien, T.K.; deVries,P.; Khanh, N.X.; Koopmans, R.; Binh, L.N.; Duc, D.D.; Kager, P.A.; van Boxtel, C.J. (1997. Anitmicrobial Agents Chemother. 41, 1069-1072
